# Telemedicine for management of patients with amyotrophic lateral sclerosis through COVID-19 tail

**DOI:** 10.1007/s10072-020-04783-x

**Published:** 2020-10-06

**Authors:** Alessandro Bombaci, Gianmarco Abbadessa, Francesca Trojsi, Letizia Leocani, Simona Bonavita, Luigi Lavorgna, on behalf of the Digital Technologies, Web and Social Media Study Group of the Italian Society of Neurology, Gioacchino Tedeschi, Giovanni Mancardi, Alessandro Padovani, Marinella Clerico, Francesco Brigo, Roberta Lanzillo, Antonio Russo, Bruno Giometto, Giulia Straccia, Rosa Iodice, Sebastiano Bucello, Pietro Annovazzi, Marcello Moccia, Luca Prosperini, Maria Laura Stromillo, Anna Maria Repice, Giuseppina Miele, Alberto Lerario, Antonio De Martino, Francesco Iodice, Francesco Di Lorenzo, Luca Cuffaro, Michele Romoli, Marcello Silvestro, Carlo Alberto Artusi

**Affiliations:** 1 “Rita Levi Montalcini” Department of Neuroscience, University of Torino, Turin, Italy; 2 Department of Advanced Medical and Surgical Sciences, II Clinic of Neurology, University of Campania “Luigi Vanvitelli”, Naples, Italy; 3 Digital Technologies, Web and Social Media Study Group of the Italian Society of Neurology, Via Pansini 5, 80131 Naples, Italy; 4 Department of Advanced Medical and Surgical Sciences, I Clinic of Neurology, University of Campania “Luigi Vanvitelli”, Naples, Italy; 5 Department of Neurorehabilitation, IRCCS San Raffaele Hospital, Milan, Italy; 6 University Vita-Salute San Raffaele, Milan, Italy

**Keywords:** Telemedicine, Teleneurology, Tele-health, Remote monitoring, ALS patients, COVID-19

## Abstract

Over the last months, due to coronavirus disease (COVID-19) pandemic, containment measures have led to important social restriction. Healthcare systems have faced a complete rearrangement of resources and spaces, with the creation of wards devoted to COVID-19 patients. In this context, patients affected by chronic neurological diseases, such as amyotrophic lateral sclerosis (ALS), are at risk to be lost at follow-up, leading to a higher risk of morbidity and mortality. Telemedicine may allow meet the needs of these patients. In this commentary, we briefly discuss the digital tools to remotely monitor and manage ALS patients. Focusing on detecting disease progression and preventing life-threatening conditions, we propose a toolset able to improve ALS management during this unprecedented situation.

Coronavirus disease 2019 (COVID-19) has been spreading throughout the world, and, on 11th of March, the World Health Organization declared it to be pandemic [1]. Healthcare systems have suddenly faced an enormous and complete rearrangement of resources and spaces, with the realization of wards entirely devoted to COVID-19 patients. Most of visits for chronic diseases have been canceled, postponed, or converted to teleconsultations (remote consultations between patients and clinicians) [2–4]. In the next months, this situation will probably persist. In this context, patients affected by chronic neurological diseases, such as amyotrophic lateral sclerosis (ALS), are at risk of being lost at follow-up with a consequently higher morbidity and mortality.

ALS is a neuromuscular progressive disorder, characterized by limb and bulbar muscle wasting and weakness. Thirty percent of patients present a bulbar onset, while 70% a spinal onset, although most of them develop bulbar impairment during the course of the disease [5]. Nowadays, there is still no curative treatment for ALS, and palliative care and symptomatic treatment are therefore essential components in the management of these patients. Death occurs in 3–5 years, generally due to respiratory paralysis [5–7]. Neurological examination and ALS Functional Rating Scale revised (ALSFRSr) are the most important tools to monitor disease progression. Early detection of severe symptoms, such as dysphagia and respiratory impairment, reduces the risk of developing *ab ingestis* pneumonia and respiratory insufficiency, improving the prognosis [8]. Therefore, it is important to establish an efficient service of telemedicine to replace face-to-face visits, monitor progression of the disease, and manage complications as soon as possible [2], especially life-threatening ones. Herein, we briefly review the available instruments to remotely manage ALS patients with the aim of proposing a digital toolset (Fig.1) to face the current imposed stay-home policy.

**Fig. 1:**
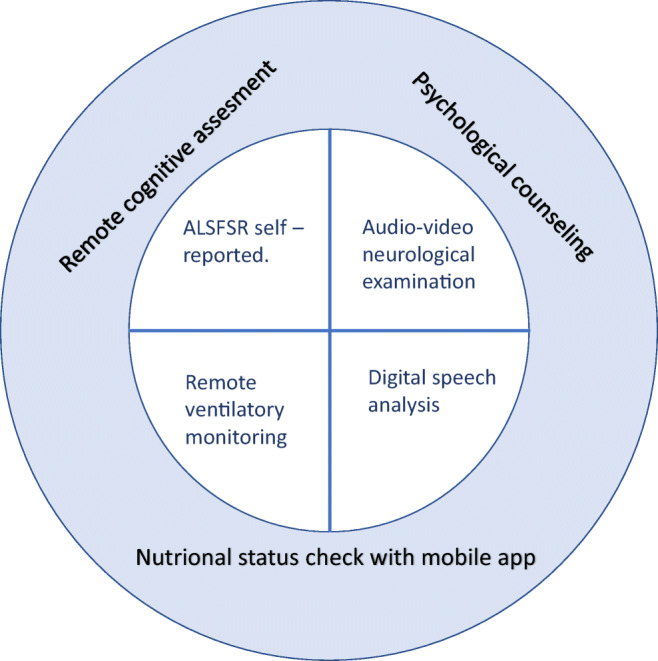
Digital tool set for ALS management in remote during COVID-19 pandemic

ALSFRSr is commonly used to evaluate ALS progression. Some recent studies explored the possibility of an online self-administered version of ALSFRSr scales [9, 10] reporting a high inter-rater and intra-rater reproducibility, and a low variability. Beyond ALSFRSr that can be easily compiled also by phone calls, performing a neurological examination remotely is also fundamental in the management of ALS. Recently, the American Academy of Neurology (AAN) published recommendations for improving a telemedicine service and suggested tools to perform a general neurological examination remotely [11]. Neurological examination through an audio-video link showed good results in terms of reliability and validity with bedside examination [12, 13], although with some limitations in the assessment of muscle tone, vibration sensation, and deep tendon reflexes.

In order to overcome limits intrinsic to remote neurological examination, digital complementary tools have been proposed. Some sensors were developed to objectively home-monitoring ALS patients. Recent pivotal studies have investigated the feasibility of wearable devices equipped with an accelerometer for motor activity assessment and heart rate variability detection [14]. Remotely monitoring invasive and non-invasive ventilation through videoconferencing or home-based self-monitoring has strong application in telemedicine and in these patients could effectively reduce morbidity and mortality [15]. Even bulbar function could be monitored in remote, through the analysis of the recordings of patients reading aloud a short paragraph, as Allison et al. reported [16]. They observed that the longitudinal evaluation of the percentage pause time expressed in seconds (a marker of speech fluency) is one of the most important markers of pre-clinical bulbar involvement in ALS. Another issue to be addressed is the monitoring of the nutritional status, which is a relevant prognostic factor. Countless nutrition-based mobile health (mHealth) applications are available for both Android and iOS. All are accessible and easy to use, but only few were assessed in clinical studies. In a recent randomized trial comparing remote nutritional counseling with or without mobile health technology in ALS patients, Nu Planit application was an acceptable and useful mobile app to check nutritional status [17]. It is a nutrition-based mHealth applications that facilitate frequent reminders and measurements. Patients can record their food habits and home weight measurements. Moreover, based on patients’ gain or loss weight, the nutritionist could remotely access to the app and modify dietary recommendation. In ALS evaluation of cognitive profile is important because around 30–50% of patients develop cognitive impairment. A review highlighted the usefulness of some neuropsychological test administered by videoconference in new diagnosis and in follow-up [18]. Unfortunately, only few cognitive tests commonly used in ALS were tested, with a good agreement between videoconference and in clinic neuropsychological test. Receiving a diagnosis of a deadly illness such as ALS can deeply affect the emotional, physical, and mental aspects of a patients’ life, and in these patients, also the psychological support is a key aspect of management. Transferring the meeting with the psychologist on a digital platform could be useful but not often accepted by the patients. Indeed, previous work on multidisciplinary digital management of ALS patients showed that psychological support was requested in few cases, probably due to personal refusal, embarrassment, or preference for a face-to-face contact [19].

All the issues debated point out the complexity of ALS patients’ management and the need to create multidisciplinary teams able to face the new challenges due to this novel approach to patients’ care [20]. As discussed in detail by Aghdam and colleagues, telemedicine offers a significant improvement in the organization of multidisciplinary team meetings compared with traditional settings. Indeed, it allows an improved access to and collaboration of medical experts. This results in an increased level of medical competence improving diagnosis, treatment, and patients’ follow-up irrespective of location [21]. In an ongoing project, De Marchi and colleagues theorized that satisfactory multidisciplinary care could be provided to ALS patients in their home using technology. In this stay-home forced situation, they employed some of the tools discussed above in order to prevent a greater decline of the physical and psychological functions of ALS patients [22]. Despite the relevance of this approach is yet to be proven, they aimed to detect, as soon as possible, how it can be useful to avoid the postponed disease-related complications of this devastating disease, trying to support patients and their caregivers in the best way [22].

Overall, in our opinion, monitoring everyday life and potential life-threatening aspects of the disease should be the main goal of telemedicine in ALS patients, particularly at the time of COVID-19 pandemic [23, 24]. As mentioned, ALSFRSr and the neurological examination are the most important instruments to monitor disease status. Therefore, they should be part of the set core of remote examination (Fig. 1) and applied to all patients on follow-up teleconsultation. While the digital assessment of motor functions through digital devices, such as accelerometer, is not reliable in a context in which patients are forced to remain home, on the contrary, speech analysis for bulbar function and monitoring of invasive and non-invasive ventilation could be crucial in reducing morbidity and mortality and should be part of the set core. A study of remote pulmonary function tests was conducted by the Penn State Health Hershey ALS Center with patients, caregivers, and respiratory therapists reporting high acceptability [25]. Moreover, monitoring nutritional status through mHealth applications is useful and accessible, and we suggest the use of those apps for all ALS patients. A future perspective could be the development of an app in order to achieve a complete self-assessed evaluation of nutritional status, respiratory function, motor ability, and subjective perceived health status, as was already done for patients with Pompe disease [26].

The psychological support is very important especially immediately after diagnosis for both patients and their relatives. Remote psychological support could be essential in a period in which hospital access has been reduced. Therefore, patients and caregivers with psychological needs should receive the opportunity for scheduled psychotherapy or on-demand calls by a psychologist.

In a recent paper, Vasta and colleagues described their experience of telemedicine with the management of ALS patients during the COVID-19 pandemic. In agreement with previous studies, they revealed that patients reported to be globally satisfied with respect to the telemedicine service they received, but, despite the risk of contracting SARS-CoV2 infection, most of them would have preferred an impatient visit. Overall, the satisfaction of patients and caregivers for the use of telemedicine is good, although face-to-face visits are still largely preferred. Thus, telemedicine for ALS patients should have a complementary and not a substitutive role, and it should replace the in-person care depending on circumstances and patient preferences [27].

Limitations in the use of those complementary tools include the high costs to acquire the instrumentation to be given to patients, the training of patients’ and caregivers, and the necessity of validation of some of these instrumentation and tools administered by videoconference or self-administered.

In conclusion, implementing telemedicine services for patients with ALS is necessary to allow direct clinical evaluation during COVID-19 pandemic, in order to plan the appropriate medical and nursing care, avoiding hospitalizations or urgent interventions.
